# Questionnaire-based subjective evaluation and analysis of factors influencing the effectiveness of treatment with the vacuum bell in children with pectus excavatum: a cross-sectional observational study

**DOI:** 10.3389/fped.2024.1467215

**Published:** 2024-10-07

**Authors:** Lihuan Zhou, Fang Deng, Yunfei Tian, Jiayi Lin, Guihong Yang, Yunfei Li, Jos M. Latour, Xicheng Deng

**Affiliations:** ^1^ Cardiothoracic Surgery Department, The Affiliated Children's Hospital of Xiangya School of Medicine, Central South University (Hunan Children's Hospital), Changsha, China; ^2^ Department of Nursing, The Affiliated Children’s Hospital of Xiangya School of Medicine, Central South University (Hunan Children’s Hospital), Changsha, China; ^3^ School of Nursing and Midwifery, Faculty of Health, University of Plymouth, Plymouth, United Kingdom

**Keywords:** pectus excavatum, children, vacuum bell, compliance, caregivers

## Abstract

**Background:**

Vacuum bell (VB) is a technique to treat pectus excavatum in children. Its effectiveness and influencing factors, however, remain under investigated. The aim of this study was to examine the therapeutic effect and its influencing factors of VB in children with pectus excavatum.

**Methods:**

A cross-sectional observational study was conducted. Parents of children with pectus excavatum who underwent treatment with a VB between January 2018 and December 2019 were recruited. A survey was designed based on previously related studies and delivered to the parents in September–October 2021. The therapeutic effect was analyzed using subjective experiences by parents. Factors related to effectiveness were analyzed through univariate analysis and multivariate logistic regression.

**Results:**

Of the 77 surveys distributed, 65 (84%) were returned. The mean duration of VB was 23.20 (SD 9.86) months. Caregivers rated the effect of VB treatment as moderate (41.5%), good (46.2%), excellent (12.3%). There were 39 children (60%) who had at least one pause of using VB for more than two weeks. Univariate analysis showed no significant difference between age, height, weight, duration of VB (months) and the effectiveness of VB therapy as defined by caregivers (*p* > 0.05) and significant difference were observed of the variables “complication of petechiae” (*p* = 0.034) and “device returned to manufacturer for repair” (*p* = 0.011). The multivariate logistic regression showed that the occurrence of complication petechiae (*p* = 0.046) was an influential factor for the effectiveness of VB.

**Conclusions:**

The evaluations reported by the parents suggested that the VB treatment was effective, although with varying degrees. The complication of petechiae seems an influencing factor to successful VB treatment in children with pectus excavatum. Further studies are needed to assess the long-term outcome and effect of VB and to improve the device and to reduce complications in order to enhance compliance and improve effectiveness.

## 1 Introduction

Pectus excavatum (PE), characterized by varying degrees of depression in the ribs and sternum on the anterior surface of the chest wall, is the most common major congenital anomaly, occurring in approximately one out of every 300 live births (1, 2). The PE diagnosis affects mostly male patients with an approximate 3–5:1 male to female ratio (1, 3).Children with moderate or severe PE may experience disruptions in cardiac and respiratory functions as well as reduced capacity for vigorous cardiovascular activities. Additionally, psychosocial stress related to physical appearance often arises during adolescence (4). In cases where the degree of pectus deformity does not immediately necessitate surgery, nonsurgical treatment options are required for patients (5). Furthermore, some patients may be hesitant to undergo surgery due to postoperative pain and potential risks associated with imperfect outcomes (6). The VB serves as an alternative treatment option for children with PE who do not require surgical intervention or refuse it (7).

The effectiveness and success of VB therapy in children are associated with the duration of VB usage (6). Several studies have investigated treatment compliance and factors influencing the effectiveness of VB therapy in children with PE (8–10). These studies have used clinical outcome measurements to evaluate the effect of VB treatment, reporting successful outcomes ranging from 13% to 43%. Achieving successful results in treating children with PE using VB requires active involvement from parents or primary caregivers who must fully adhere to the treatment technique. However, there is currently a gap in the literature regarding parental experiences. Therefore, it is recommended to employ a combination of subjective and objective measures for assessing therapy success (5, 11).

The objective of this study was to investigate the experiences of parents with children diagnosed with PE who underwent VB treatment, and to identify factors influencing the effectiveness of VB treatment. This manuscript adheres to the guidelines outlined in the Strengthening the Reporting of Observational Studies in Epidemiology (STROBE) Statement, ensuring comprehensive reporting of observational studies (12).

## 2 Materials and methods

### 2.1 Study design

The effectiveness of nonsurgical treatment for PE with the VB was evaluated through a cross-sectional study using questionnaires conducted at Hunan Children's Hospital in Hunan, China. This study received approval from the Institutional Ethics Committee of Hunan Children's Hospital (No. HCHLL-2024-83), and informed consent was obtained from all participants and their parents.

### 2.2 Setting

#### 2.2.1 Indications for VB therapy

The indications for VB therapy included moderate to severe PE as determined by inspection or CT scan, the Haller index (13, 14) is the ratio of the transverse (coronal) internal thoracic measurement divided by the AP (sagittal) measurement at its deepest point. Mild is categorized as <3.2. Moderate includes Haller indices of 3.2 ± 3.5. Severe encompasses all number >3.5, in cases where the patient was deemed too young for surgery, had experienced recurrence after surgery, or expressed aversion to surgical intervention. Contraindications to VB therapy were limited to coagulopathies. All potential candidates for VB treatment underwent evaluation and approval or disapproval by a single surgeon.

#### 2.2.2 Treatment protocol

**Figure 1:**
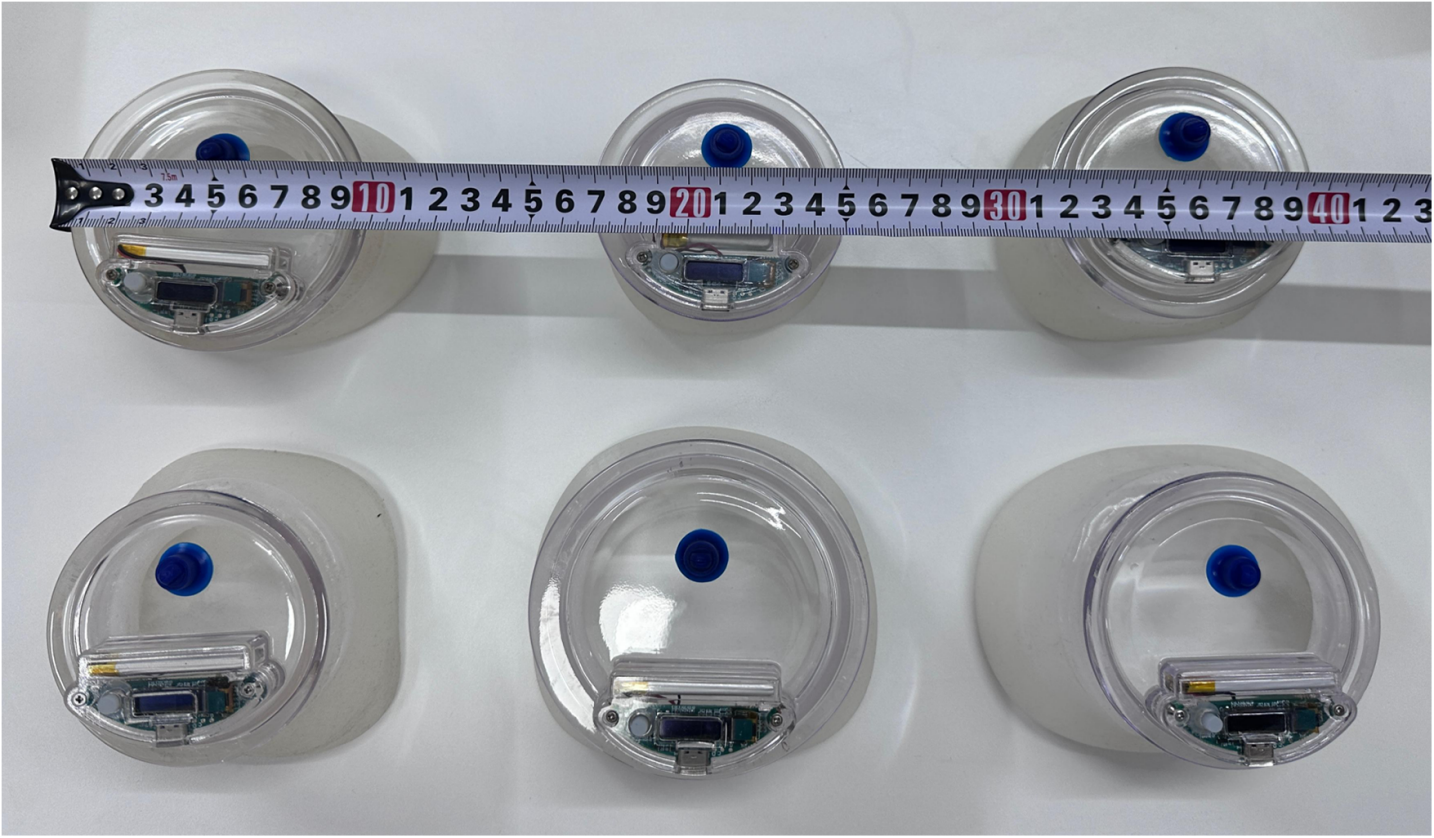
Different sizes of the VBs.

During the initial treatment with VB (Guangzhou Yikang Medical Technology Co., Ltd.) (Figure 1), patients and their parents received comprehensive training from the surgeon at our clinic. The choice of VB sizes is contingent upon the degree and scope of concavity in patients with PE. Our treatment initiation protocol involves a gradual increase in daily usage time, starting with two 10–15 min sessions per day, which progressively increases by 5–10 min per week until reaching two 60-min sessions daily. Subsequently, usage time is flexible and patients are encouraged to extend it as much as possible. The recommended vacuum pressure ranges are determined based on patient age (Table 1), while the negative pressure of VB is established according to individual tolerance levels without causing discomfort. The entire course of treatment lasts for one year and may be extended if necessary.

**Table 1:** Recommended negative pressures of different age groups.

Age	Negative pressure	
	Early period	Late period
<1 year	7 kpa–10 kpa	10 kpa–15 kpa
1–3 years	10 kpa–15 kpa	15 kpa–20 kpa
3–10 years	10 kpa–20 kpa	15 kpa–25 kpa
10–16 years	15 kpa–20 kpa	20 kpa–30 kpa
Adults	20 kpa–30 kpa	33 kpa

#### 2.2.3 Design of follow-up questionnaire

Two experts in PE independently compiled questions from previously validated questionnaires used in other deformative diseases [such as pectus carinatum (PC) and scoliosis] and developed additional items to quantitatively assess compliance with VB therapy and evaluate potential influencing factors in patients with PE. The questionnaire comprises 20 items, which measure participants' characteristics, daily VB usage (average duration in minutes and frequency), treatment duration (accumulated months of use), vacuum pressures (kPa), complications, and the effectiveness of VB therapy.

### 2.3 Participants and study size

The questionnaires were distributed to the parents between September and October 2021. Parents whose children received VB treatment from January 2018 to December 2019 were included in this study. Inclusion criteria comprised of: VB treatment for PE, age ≥1 year, treatment duration ≥6 months, and consent provided by the patient's father, mother, or guardian for accessing their data for research purposes. Exclusion criteria consisted of: absence of chest sinking determination at the start of treatment, osteogenic syndrome presence, and withdrawal from clinical follow-up.

### 2.4 Variables

The parents were invited to participate in the study and complete online questionnaires 6 months to one year after the commencement of the research. The caregivers provided reports on daily VB usage (average duration in minutes and frequency per day), treatment duration (accumulated months of use), vacuum pressures (kPa), among other factors. Apart from subjective assessment, we also used the CT scan and 3D scanner to record treatment outcomes. After approval, every patient underwent initial CT scan upon ordering of a VB and later follow-ups every 3 months to half a year during use of the device. The 3D scanner was used to acquire body surface data on the standing position, which was used to record treatment outcomes, the collected body surface data were used for deformity documentation in order to compare with follow-up scans during the course of treatment.

### 2.5 Statistical methods

The primary variables were analyzed using descriptive statistics and frequencies. Continuous data with a normal distribution were presented as mean ± standard deviation, while non-normally distributed data were reported as median and interquartile range. Categorical data were expressed in frequencies and percentages. Univariate analysis and multivariate logistic regression were conducted to analyze the therapeutic effect and identify factors associated with its effectiveness. Statistical analysis was performed using SPSS 27.0 (SPSS Inc., Chicago, IL, USA, 2020) software for Windows.

## 3 Results

### 3.1 Participants

**Figure 2:**
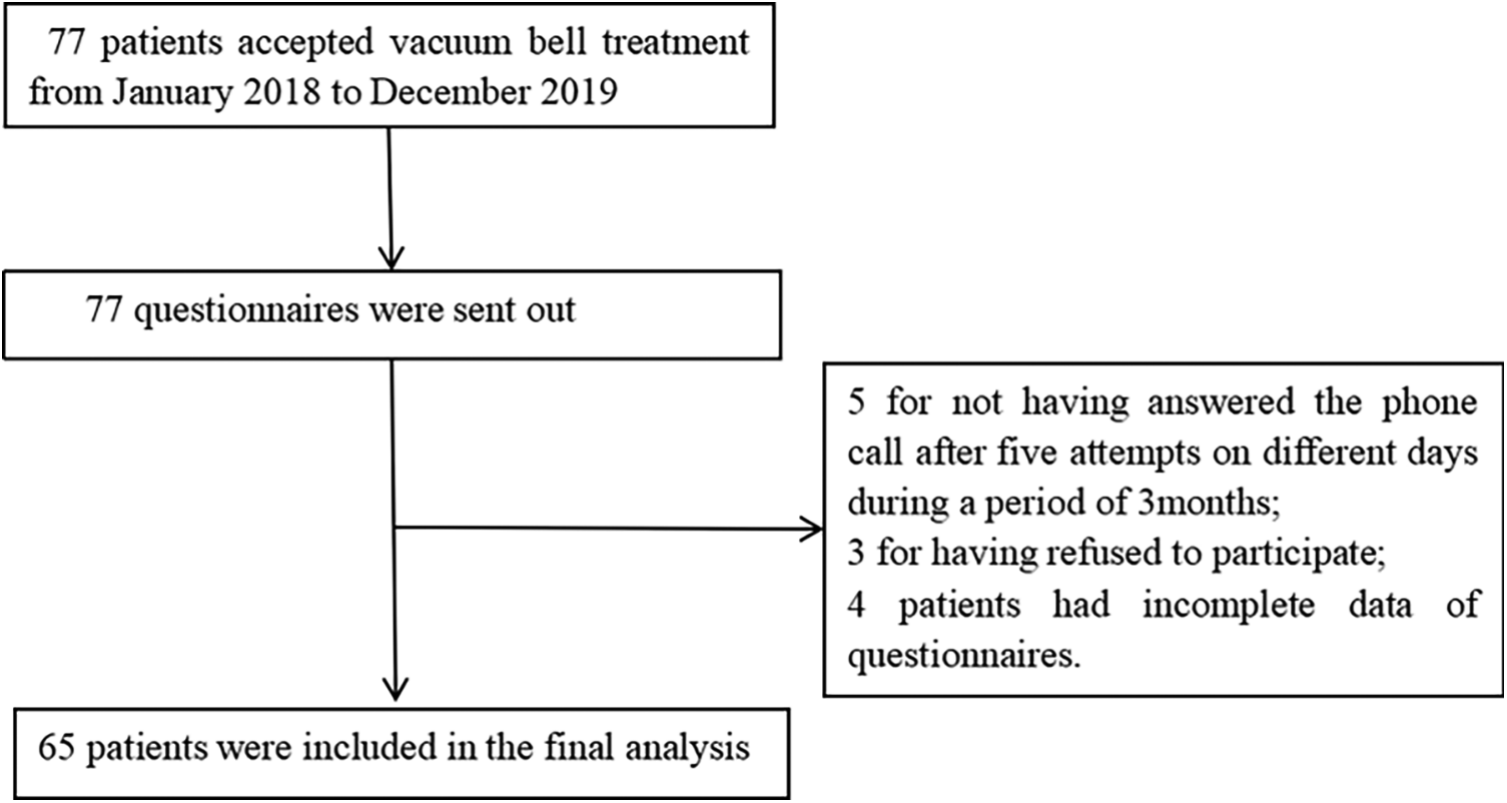
Study flow chart.

A total of 77 questionnaires were distributed, and 65 valid responses were obtained (Figure 2). In this study, a cohort of 65 children (50 males, 15 females) was enrolled. The mean age was (6.37 ± 3.28) years, the average height was (120.18 ± 20.20) cm, and the mean weight was (23.80 ± 10.22) kg (Table 2).

**Table 2:** Demographics of 65 patients.

Mean ± SD/median (IQR)/frequency, percentage	
Age, years	6.37 ± 3.28
Male	50 (77%)
Female	15 (23%)
Height, cm	120.18 ± 20.20
Weight, kg	23.80 ± 10.22
Family members with PC	9 (13.8%)
Family members with PE	2 (3.1%)

IQR, interquartile range; SD, standard deviation; PE, pectus excavatum; PC, pectus carinatum.

### 3.2 Descriptive data

The mean duration of VB usage was 23.20 ± 9.86 months. Regarding complications, petechiae was reported by 29 patients (44.6%) during VB use, while blistering was reported by 14 patients (21.5%), along with some cases of pain and/or discomfort. All symptoms resolved after a short pause in treatment, without any permanent sequelae from side effects. In terms of effectiveness, based on subjective evaluations by caregivers, the majority considered the therapeutic effect to be moderate (27 patients; 41.5%) or good (30 patients; 46.2%), while 8 patients (12.3%) rated it as excellent, resulting in an overall effective rate of 100% for VB treatment. Among the participants, 52 patients (80%) continued using the VB unless instructed otherwise to pause or terminate its use, whereas 39 patients (60%) experienced at least one pause lasting more than two weeks (Table 3).

**Table 3:** Follow-up and questionnaire results.

Mean ± SD/median (IQR)/frequency, percentage	
Questionnaire responders	65 (84.4%)
How was the device used?	
Duration per time, minute	
15	1 (1.1%)
30	34 (37%)
45	9 (9.8%)
60	15 (16.3%)
90	3 (3.3%)
120	3 (3.3%)
Times per day	
1	19 (20.7)
2	45 (48.9)
3	1 (1.1%)
Vacuum, kPa	
0	8 (12.3%)
7–10	12 (18.5%)
10–15	12 (18.5%)
15–20	26 (40%)
20–30	13 (20%)
Complications	
Petechia	29 (44.6%)
Blistering	14 (21.5%)
Discomfort	4 (6.2%)
Pain	10 (15.4%)
Others	5 (7.7%)
Effectiveness by questionnaire	
Poor	0
Moderate	27 (41.5%)
Good	30 (46.2%)
Excellent	8 (12.3%)
Duration, months	23.20 ± 9.86

IQR, interquartile range; SD, standard deviation.

### 3.3 Outcome data

#### 3.3.1 The results of variance analysis

The analysis of variance revealed no statistically significant differences (*P* > 0.05) in age, height, weight, duration of usage (months) and the effectiveness of VB therapy. The results of univariate analysis showed that there were no significant differences (*P* > 0.05) between the effectiveness of VB therapy and the frequency of use per day, treatment duration for each use and the vacuum pressure. However, significant differences were observed (*P* < 0.05) in the occurrence of petechiae during VB treatment and the need to return to the manufacturer for repair (Table 4).

**Table 4:** Univariate analysis of the factors for effectiveness of the VB therapy.

Variable	Effectiveness of the VB Therapy			*F*/χ^2^	*P*
	Moderate (*n* = 27)	Good (*n* = 30)	Excellent (*n* = 8)		
Age	6.26 ± 4.11	6.47 ± 2.60	6.38 ± 2.77	0.028	0.973
Height	119.07 ± 25.62	121.07 ± 16.01	120.63 ± 14.93	0.069	0.933
Weight	24.44 ± 13.27	23.74 ± 7.96	21.88 ± 5.77	0.191	0.827
Duration of VB usage(months)	23.30 ± 11.52	22.90 ± 9.16	24.00 ± 6.93	0.04	0.961
Duration per time, minute				11.395	0.328
15 min	0 (0.00)	1 (100.00%)	0 (0.00)		
30 min	14 (41.18%)	14 (41.18%)	6 (17.65%)		
45 min	3 (33.33%)	5 (55.56%)	1 (11.11%)		
60 min	7 (46.67%)	8 (53.33%)	0 (0.00)		
90 min	0 (0.00)	2 (66.67%)	1 (33.33%)		
120 min	3 (100.00)	0 (0.00)	0 (0.00)		
Vacuum, kPa				9.173	0.328
0 KP	5 (62.50%)	3 (37.50%)	0 (0.00)		
7 KP–10 KP	3 (50.00%)	3 (50.00%)	0 (0.00)		
10 KP–15 KP	7 (58.33%)	3 (25.00%)	2 (16.67%)		
15 KP–20 KP	6 (23.08%)	15 (57.69%)	5 (19.23%)		
20 KP–30 KP	6 (46.15%)	6 (46.15%)	1 (7.69%)		
Complications					
Petechia	7 (24.10%)	18 (62.10%)	4 (13.80%)	6.784	0.034
Blistering	3 (21.40%)	10 (71.40%)	1 (7.10%)	4.594	0.101
Discomfort	3 (75.00%)	1 (25.00%)	0 (0.00)	2.087	0.352
Pain	4 (40.00%)	5 (50.00%)	1 (10.00%)	0.096	0.953
Others	2 (40.00%)	3 (60.00%)	0 (0.00)	0.895	0.0639
Reasons of pause of use more than 2 weeks					
Device return to the manufacturer for repair	2 (33.30%)	1 (16.70%)	3 (50.00%)	8.983	0.011

VB, vacuum bell.

#### 3.3.2 The results of multivariate logistic regression

The effect of VB therapy after treatment was assessed as the dependent variable, while the occurrence of petechiae during the VB treatment process and equipment return for repair were considered as independent variables in a multivariate linear regression analysis. Based on the *F*-test results (*F* = 3.717, *p* = 0.030 < 0.05), it can be concluded that model construction is statistically significant. The R-squared value was 0.107, indicating that the combined influence of petechiae occurrence during VB treatment and equipment return for repair explains 10.7% of the variation in post-VB treatment effect evaluation (Table 5). Analysis from the regression coefficient reveals that petechiae occurrence during VB treatment significantly affects post-treatment effect evaluation (*P* < 0.05). Therefore, assessment of VB therapy effectiveness primarily relies on monitoring petechiae occurrence during treatment (Table 6).

**Table 5:** ANOVA^a^.

Model		Sum of square	DOF	MS	*F*	Sig.
1	Regression	3.152	2.000	1.576	3.717	.030^b^
	Residual error	26.294	62.000	0.424		
	Total	29.446	64.000			

^a^ Dependent variable: Evaluation of the effectiveness of suction therapy.

^b^ Predictor variables: (constant), returned to the manufacturer for repair, VB treatment with or without petechiae occurring during the process.

ANOVA, analysis of variance.

**Table 6:** Multivariate logistic analysis results on influencing factors of the VB therapy.

Variable	B	S.E	β	*t*	*P*
Petechiae occurred during treatment	0.331	0.163	0.245	2.037	0.046
Device return to the manufacturer for repair	0.486	0.279	0.209	1.741	0.087

*F* = 3.717, *p* = 0.030 < 0.05, *R*^2^ = 0.107, Adjusted R-squared = 0.078.

## 4 Discussion

### 4.1 Key findings

The objective of our study was to investigate the therapeutic efficacy and influencing factors of VB therapy in pediatric patients with PE. Our findings demonstrate a 100% effective rate for VB therapy, with an average duration of vacuum bell usage being (23.20 ± 9.86) months. Patients who experienced reduced incidence of petechiae complications were more likely to achieve favorable treatment outcomes, suggesting that proactive complication prevention and intensive management may be necessary in these individuals.

The non-surgical conservative treatment of PE with VB therapy was initially described by Schier et al. in 2005 (6). Since then, it has gained increasing popularity as a viable alternative to surgical intervention for carefully selected patients (15–17). However, the efficacy of VB therapy heavily relies on patient compliance and motivation. Therefore, it is crucial to conduct rigorous follow-up assessments to evaluate the long-term stability of correction achieved through VB therapy (17). Obermeyer (10) reported that nearly one third of their patients were either lost to follow-up or exhibited poor compliance.

In our study, all patients expressed satisfaction with the use of the VB, although subjective assessment of improvement in PE varied among individuals. The subjective evaluation questionnaire revealed a 100% effective rate for VB therapy, with 41.5% showing moderate correction, 46.2% showing good correction, and 12.3% showing excellent correction. These findings are consistent with Haecker's (18) results from a study involving 133 patients, where sternum flattening was observed in 79%, despite a treatment discontinuation rate of 10%. However, the effectiveness rate in our study was significantly higher than the reported range of successful corrections in literature (13.5%–43.6%) (9, 10, 18). This discrepancy may be attributed to variations in evaluation tools used; some studies employed absolute depression depth measurements that did not account for patient body size or necessitated specialized measurement tools to quantify correctness, while others utilized Haller Index, Correction Index or modified percentage depth (19). In our questionnaire-based assessment conducted by caregivers who monitored long-term home treatments without hospital visits or during review intervals, treatment effectiveness was subjectively evaluated based on improvements in the degree of depression, though this approach is subjective it does provide insights into treatment progress and efficacy.

Contrary to previous literature, this study found no statistically significant association between age at treatment and outcomes. Obermeyer et al. reported better results in children under 11 years of age, possibly due to inclusion of both adult and pediatric patients (10). Therefore, the variation in complete correction rates among different age groups may be attributed to differences in chest wall flexibility. Our study included children with a mean age of (6.37 ± 3.28) years, all younger than 10 years. As children grow older, chest wall becomes stiffer and less flexible, which can influence treatment outcomes. All patients with available follow-up data demonstrated improved chest appearance as objectively and subjectively confirmed. Thus, the impact of age on the treatment outcome was limited in our study.

The effects of height, weight, duration of usage (months), duration per time and negative pressure on the treatment outcome were not statistically significant. This finding is consistent with Dengke Luo (8), who reported that daily usage time, initial negative pressure, sex, comorbidities, and family history also did not show statistical significance in relation to the treatment outcome.

The identification of influencing factors on treatment outcomes in this study has direct clinical applicability. Side effects, such as petechiae, blistering, discomfort or pain were reported at a similar rate to that found in literature. The occurrence of side effects may be attributed to excessive negative pressure regulation; however, all observed side effects were reversible and resolved by reducing suction pressure or pausing treatment for no more than two weeks (20). The utilization of VB was discontinued due to the occurrence of complications, which had the potential to impact treatment efficacy. This highlights the importance of educating patients and their families on managing complications throughout the entire treatment process, such as providing one-to-one guidance via Wechat, which proves to be an effective approach.

Another factor influencing therapeutic efficacy is equipment maintenance, which involves returning it to the manufacturer. As VB therapy is relatively new in the hospital where the study was conducted, there may be instances of equipment damage due to factors such as device malfunctions or improper use during treatment. Repairing the equipment requires time for it to be sent back and forth from the manufacturer, potentially affecting treatment effectiveness. In future treatments, we will establish communication with the manufacturer to ensure strict control over VB quality, thereby reducing product quality issues and minimizing treatment interruptions caused by objective reasons.

### 4.2 Limitations

The limitations of this study include its retrospective design and the presence of patients lost to follow-up or with incomplete data. Additionally, it is important to note that the survey subjects were excluded from a single tertiary pediatric hospital, resulting in a relatively small sample size. Moreover, the self-reported questionnaire data on daily usage and pressure may be susceptible to memory-related or other errors. Furthermore, due to the young age of our patients and their exposure to winter during treatment, several pauses occurred for weeks or even months. Although parents were required to report these pauses, there is a possibility of errors when calculating the total duration. Furthermore, while treatment outcomes were assessed based on caregivers' measurements, aspects such as quality of life and changes in self-belief were not evaluated. Future studies should incorporate patient evaluations of outcomes and measure chest depression.

## 5 Conclusion

The subjective evaluations reported by parents indicated the clear effectiveness of VB, although with varying degrees. It is imperative to enhance the product and mitigate complications in order to optimize compliance and further improve its efficacy. Additionally, conducting subsequent follow-up studies is essential for evaluating the effectiveness of this therapeutic tool.

## Data Availability

The raw data supporting the conclusions of this article will be made available by the authors, without undue reservation.
